# Expression profiles and functions of ferroptosis-related genes in the placental tissue samples of early- and late-onset preeclampsia patients

**DOI:** 10.1186/s12884-022-04423-6

**Published:** 2022-01-31

**Authors:** Nana Yang, Qianghua Wang, Biao Ding, Yingying Gong, Yue Wu, Junpei Sun, Xuegu Wang, Lei Liu, Feng Zhang, Danli Du, Xiang Li

**Affiliations:** 1 Reproductive Medicine Center, Department of Obstetrics and Gynecology, The First Affiliated Hospital of Bengbu Medical College, Bengbu, 233004 Anhui China; 2 Anhui Province Key Laboratory of Immunology in Chronic Diseases, The First Affiliated Hospital of Bengbu Medical College, Bengbu, 233004 Anhui China

**Keywords:** Preeclampsia, Early-onset preeclampsia, Ferroptosis, Bioinformatics

## Abstract

**Background:**

The accumulation of reactive oxygen species (ROS) resulting from upregulated levels of oxidative stress is commonly implicated in preeclampsia (PE). Ferroptosis is a novel form of iron-dependent cell death instigated by lipid peroxidation that likely plays an important role in PE pathogenesis. This study aimed to investigate the expression profiles and functions of ferroptosis-related genes (FRGs) in early-onset preeclampsia (EOPE) and late-onset preeclampsia (LOPE).

**Methods:**

Gene expression data and clinical information were downloaded from the Gene Expression Omnibus (GEO) database. The “limma” R package was used to screen differentially expressed genes. Gene Ontology (GO), Kyoto Encyclopedia of Genes and Genomes (KEGG) and protein–protein interaction (PPI) network analyses were conducted to investigate the bioinformatics functions and molecular interactions of significantly different FRGs. Quantitative reverse transcription polymerase chain reaction (RT-qPCR) was used to verify the expression of hub FRGs in PE.

**Results:**

A total of 4215 differentially expressed genes (DEGs) were identified between EOPE and preterm cases while 556 DEGs were found between LOPE and term controls. Twenty significantly different FRGs were identified in EOPE subtypes, while only 3 FRGs were identified in LOPE subtypes. Functional enrichment analysis revealed that the differentially expressed FRGs were mainly involved in EOPE and enriched in hypoxia- and iron-related pathways, such as the response to hypoxia, iron homeostasis and iron ion binding process. PPI network analysis and verification by RT-qPCR resulted in the identification of the following five FRGs of interest: *FTH1*, *HIF1A*, *FTL*, *MAPK8* and *PLIN2*.

**Conclusions:**

EOPE and LOPE have distinct underlying molecular mechanisms, and ferroptosis may be mainly implicated in the pathogenesis of EOPE. Further studies are necessary for deeper inquiry into placental ferroptosis and its role in the pathogenesis of EOPE.

**Supplementary Information:**

The online version contains supplementary material available at 10.1186/s12884-022-04423-6.

## Background

Preeclampsia (PE) is a clinical syndrome characterized by gestational hypertension and proteinuria with maternal end-organ damage, which occurs after 20 weeks of gestation.

It occurs in 5–7% of pregnancies and is a leading cause of maternal and perinatal mortality [1]. PE can be classified into two categories by the time of onset of clinical signs and symptoms [2, 3]: early-onset preeclampsia (EOPE, < 34 weeks of gestation) and late-onset preeclampsia (LOPE, ≥34 weeks of gestation). EOPE has more severe manifestations and complications than LOPE. It is widely accepted that EOPE is mainly due to abnormal implantation and placentation in early gestation, whereas LOPE commonly results from placental dysfunction caused by maternal disease [4].

Generally, PE is considered a two-stage disease [4, 5]. Stage 1 is composed of abnormal implantation and malplacentation, while stage 2 is the clinical syndrome that results from the release of factors by a dysfunctional placenta. Local hypoxia and ischemia caused by placental maldevelopment are powerful inducers of oxidative stress [6]. Oxidative stress stimulates the release of antiangiogenic factors, proinflammatory cytokines and soluble endoglin into the maternal circulation, which may result in maternal endothelial dysfunction, the inflammatory response and hypertension [7–10]. Although oxidative stress is proposed to be implicated in the clinical manifestations of PE, the underlying mechanism remains largely unknown.

Ferroptosis is a novel form of iron-dependent cell death that is quite different from apoptosis, necrosis, and autophagy in terms of its morphology, biochemistry, and genetics [11]. It is instigated by the accumulation of iron-dependent hydroxyperoxidized phospholipids [11]. Ferroptosis has recently become a key focus of research in multiple diseases, including brain injury, heart injury, acute renal failure, asthma, and cancer [12–14]. Recent studies have suggested that ferroptosis might play important roles in the placental pathogenesis of PE [15–17]. However, to our knowledge, few studies have systematically analyzed ferroptosis in PE and its clinical subtypes. In the present study, firstly, we analyzed the expression profiles in the placental tissues of patients with EOPE and LOPE. Furthermore, the expression of ferroptosis-related genes (FRGs) was comprehensively investigated, and hub FRGs were identified in EOPE through bioinformatics analysis. We found, for the first time, that many key proteins implicated in the regulation of ferroptosis were aberrantly expressed in the placental tissues of patients with EOPE, but few were aberrantly expressed in the placental tissues of patients with LOPE. These results highlight the critical roles of ferroptosis in EOPE, which would be helpful for further elucidation of ferroptosis-related molecular mechanisms in PE pathogenesis.

## Methods

### Acquisition of gene expression data

The gene expression profiling dataset GSE74341, based on the GPL16699 Agilent-039494 SurePrint G3 Human GE v2 8x60K Microarray platform, was downloaded from the Gene Expression Omnibus (GEO) database (https://www.ncbi.nlm.nih.gov/geo/). The experiment contained 25 samples consisting of placental tissues from patients with early-onset (*n* = 7; gestational age at delivery < 34 weeks), and late-onset (*n* = 8; gestational age at delivery > 36 weeks) PE and controls who delivered preterm (*n* = 5; gestational age at delivery < 34 weeks) or at term (*n* = 5; gestational age at delivery > 36 weeks). There was no need for patient consent or ethics committee approval, since all the information on gene expression and samples were downloaded from public databases.

### Differentially expressed genes

The differentially expressed genes (DEGs) were identified using the “limma” R package. The cutoff values were determined according to the parameters of an adjusted *P* value < 0.05. To obtain the significantly differentially expressed FRGs in the placental tissues of patients with EOPE, DEGs in the comparisons of patients with EOPE and preterm controls were determined by the criteria of the adjusted *P* value < 0.05 and log2-fold change > 1.

### Gene ontology (GO) terms and pathway enrichment analysis for FRGs of EOPE patients

Ferroptosis-related gene sets were acquired from FerrDb (http://www.zhounan.org/ferrdb/index.html) [18]. GO [19] and Kyoto Encyclopedia of Genes and Genomes (KEGG) [20] analyses were performed by R software. They were used for the functional enrichment analysis of FRGs, including biological processes (BPs), cellular components (CCs), molecular functions (MFs) and pathway analyses. The Benjamini-Hochberg method was used to adjust the *P* values. Adjusted *P* values < 0.05 were set as the threshold values.

### Gene cluster identification and protein–protein interaction (PPI) network analysis

The STRING database (https://string-db.org/) was used for the PPI network analysis to obtain a protein network interaction diagram [21]. The result was downloaded from the online database of STRING and then imported into Cytoscape v3.8.0 software to select the key nodes for visualizing the molecular interaction networks. The CytoHubba plugin was used to identify the hub genes from the PPI network.

### Quantitative reverse transcription polymerase chain reaction (RT-qPCR)

All placental samples were collected with permission from the Ethical Committee of the First Affiliated Hospital of Bengbu Medical College (2021KY036), and informed written consent was obtained from the patients. PE patients were diagnosed based on the guidelines of the American College of Obstetrics and Gynecology (ACOG) [22]. Briefly, the detailed criteria for PE were new-onset hypertension (≥ 140/90 mmHg) on at least 2 occasions that were at least 4 h apart, accompanied by one or more of the following features: proteinuria (≥ 300 mg/24 h), maternal organ dysfunction (including renal, hepatic and neurological), hematological involvement and uteroplacental dysfunction. EOPE were defined as PE patients who have clinical signs and symptoms before 34 weeks of gestation (between 20 and 34 completed gestational weeks). Pregnant women with cardiovascular diseases, metabolic syndrome, endocrine diseases, liver or kidney diseases or fetuses with malformations or chromosomal abnormalities were excluded. Placental tissues (1 × 1 × 1 cm^3^) were obtained from the center of the umbilical cord immediately after delivery. The tissues used for RT-qPCR were frozen at − 80 °C after being washed with a balanced salt solution to remove blood.

Total RNA from placental tissues was extracted using TRIzol (15,596,018, Invitrogen, Carlsbad, Calif). The concentration and purity of the extracted RNA were assessed by NanoDrop™ One/OneC (Thermo Fisher Scientific). Reverse transcription was implemented using a NovoScrip® Plus All-in-one 1st Strand cDNA Synthesis Kit (E042-01B, Novoprotein, China) at 42 °C for 5 min, 50 °C for 15 min, and finally, at 75 °C for 5 min. PCRs were performed on NovoStart® SYBR qPCR SuperMix Plus (E167-01A, Novoprotein, China). The primer sequences are shown in S1 Table.

### Statistical analysis

All statistical analyses were presented as the means ± SEM. R software (version 4.0.2) and GraphPad software were used to analyze the data. The sample size was estimated to provide a power of 90% to reject the null hypothesis of equal means using a 2-sided, 2-sample equal-variance t test. Continuous values and count data were analyzed using t-tests and chi-squared tests, respectively. A *P* value < 0.05 was considered statistically significant.

## Results

### Differentially expressed genes in the placentas of patients with PE and PE subtypes

**Fig. 1:**
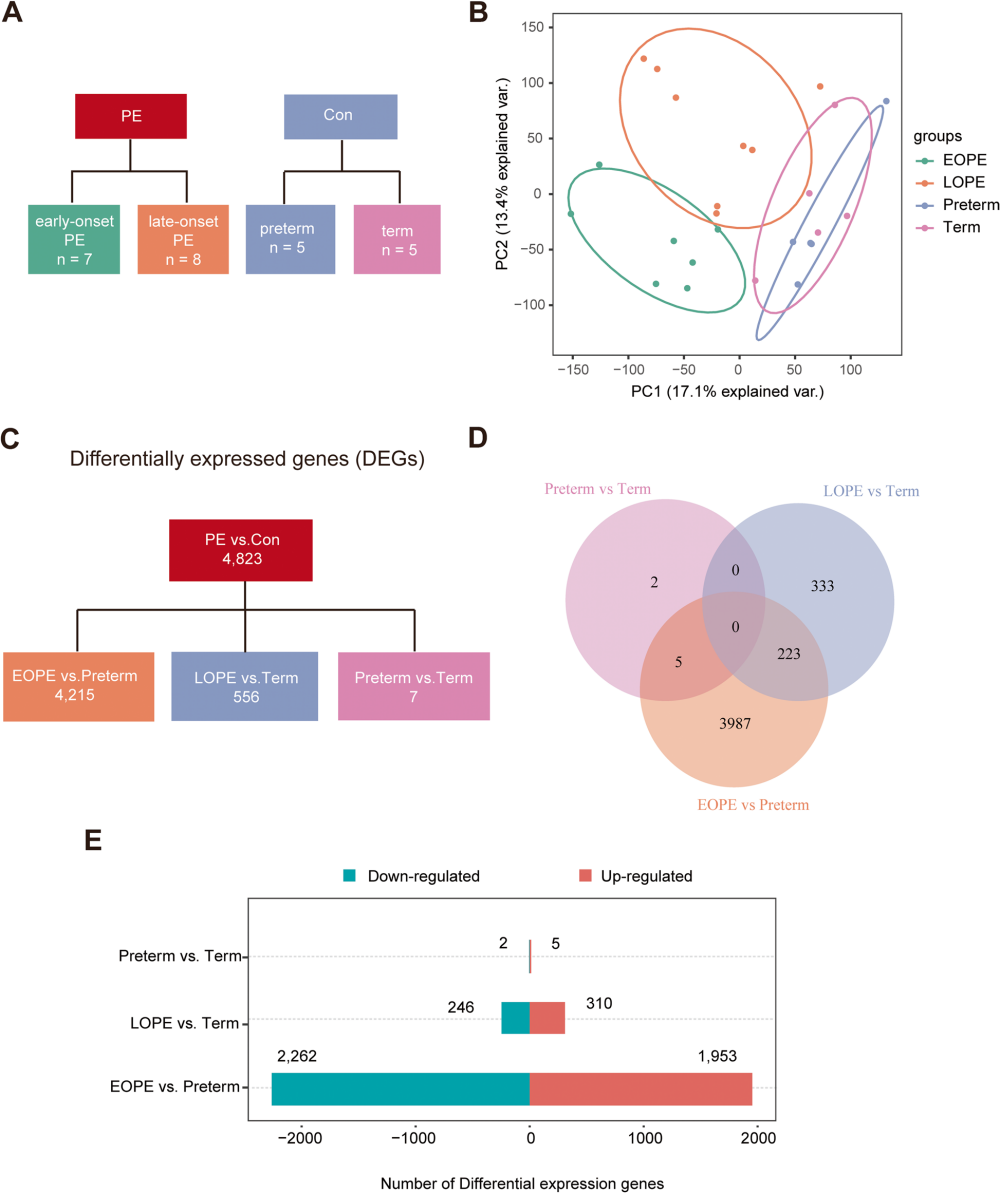
Differentially expressed genes in the placental samples of PE patients. **A** Clinical subtypes of PE patients and controls who delivered preterm or at term. **B** The principal component analysis (PCA) of the gene expression datasets. **C** The differentially expressed genes (DEGs) for the different comparisons. **D** The overlapping genes between the comparisons of EOPE and LOPE patients, LOPE patients and preterm controls, preterm and term controls and EOPE patients and term controls. **E** Numbers of up- and downregulated genes in the comparisons

The microarray expression in placental tissues from patients with EOPE, LOPE, and preterm and at term controls was downloaded from dataset GSE74341 in the GEO database (Fig. 1A). To explore sample features in gene expression, principal component analysis (PCA) was performed on the downloaded dataset. The results from the PCA showed that the EOPE samples were clustered together and separated from the LOPE subtypes and nonPE samples (Fig. 1B). The LOPE samples were also separated from nonPE placental samples.

The DEGs between the placental tissues from EOPE, LOPE, preterm and term controls were analyzed using the limma R package. DEGs were determined by the criteria of an adjusted *P* value < 0.05. A total of 4215 DEGs were identified between EOPE and preterm controls, while only 556 DEGs were found between LOPE and term cases (Fig. 1C). As shown in Fig. 1D, 223 DEGs were observed in both EOPE and LOPE. In addition, there were more downregulated than upregulated genes in the comparisons of EOPE patients and preterm controls (Fig. 1E).

### The differential expression of FRGs in the PE and PE subtypes

**Fig. 2:**
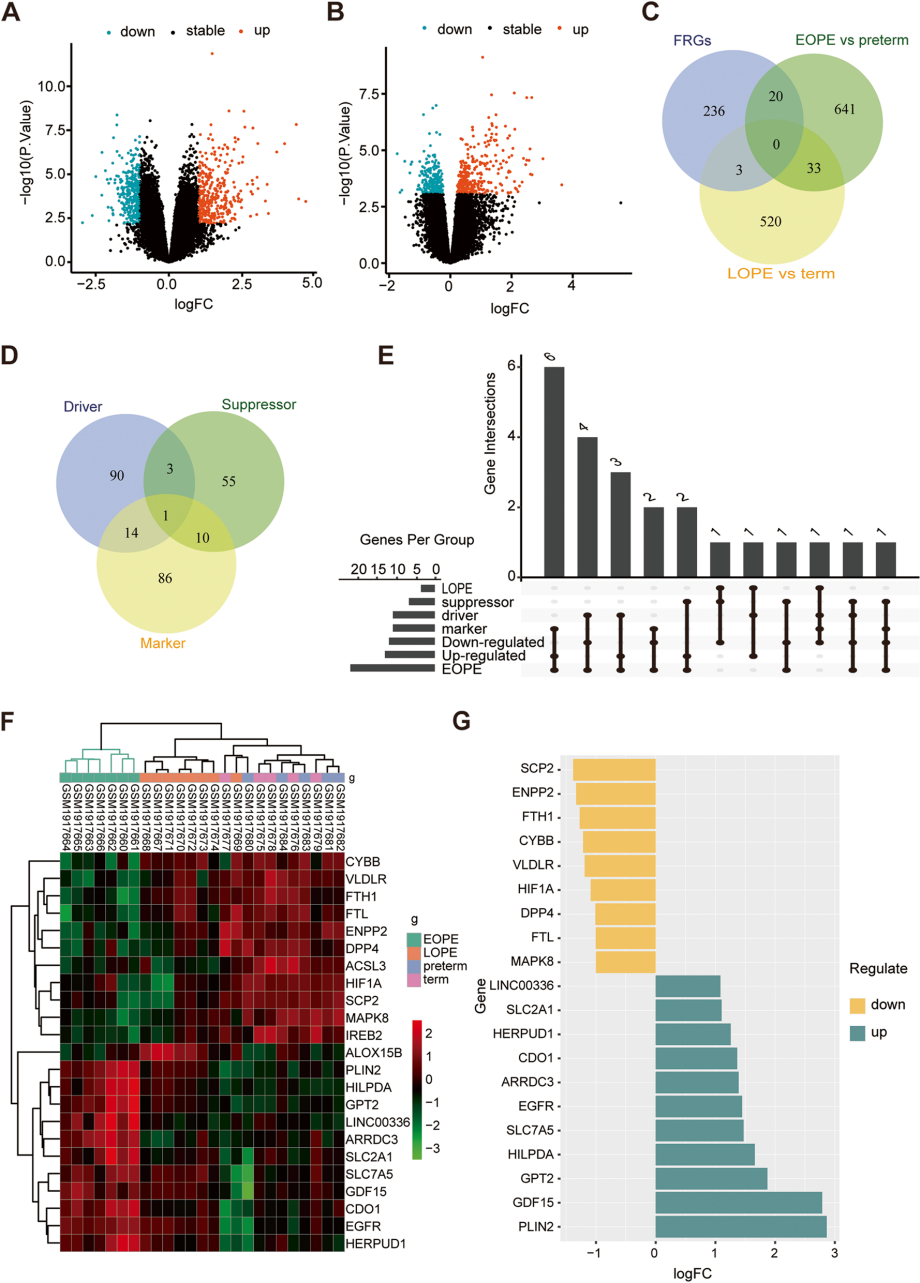
Differentially expressed FRGs in the placental samples of PE patients. **A** The volcano plot of differentially expressed genes in the placental samples of EOPE patients. **B** The volcano plot of differentially expressed genes in the placental samples of LOPE patients. **C** The overlapping genes between FRGs and comparisons of EOPE patients and preterm controls and LOPE patients and term controls. **D** The overlapping FRGs between drivers, suppressors and markers. **E** The distribution of up- and downregulated ferroptosis regulators and markers in EOPE and LOPE patients. **F** The heatmap of differentially expressed FRGs in the placental samples of preeclampsia patients and the dendrogram based on the clustering analysis. Green represents downregulation, while red represents upregulation of the genes. **G** Deviation plot of up- and downregulated FRGs in the placental samples of EOPE patients

To avoid the impact of the imbalance in the number of DEGs on the inclusion of FRGs, the criteria for determining DEGs in comparisons of EOPE patients and preterm controls were determined as follows: an adjusted *P* value < 0.05 and a log_2−_fold change > 1. As shown in the volcano plot in Fig. 2A and B, there were similar numbers of DEGs in EOPE and LOPE patients. After intersection with FRGs, 20 differentially expressed FRGs were found between EOPE and preterm samples, while only 3 were found between LOPE and term samples (Fig. 2C and Table 1). A total of 259 FRGs were downloaded from FerrDb, including drivers, suppressors and markers promoting, preventing and indicating the occurrence of ferroptosis, respectively (Fig. 2D and Table S2). As shown in Fig. 2E, almost half of the FRGs (45%, 9/20) in the placentas of EOPE patients were markers that indicated ferroptosis occurrence. The clustering analysis of significantly different FRGs showed that the EOPE samples were closely clustered together (Fig. 2F). There were 9 down- and 11 up-regulated FRGs in placenta of EOPE patients (Fig. 2G and Table 1).

**Table 1:** The differentially expressed FRGs in placenta of EOPE patients

Upregulated			Downregulated		
Gene symbol	Adjusted ***P*** value	logFC	Gene symbol	Adjusted ***P*** value	logFC
**DRIVER**					
EGFR	6.64E-03	1.447	IREB2	8.71E-03	0.289
CDO1	8.76E-03	1.365	CYBB	6.25E-03	1.215
HILPDA	2.07E-03	1.660	SCP2	5.63E-03	1.382
			DPP4	1.18E-02	1.010
			MAPK8	1.35E-04	1.000
**MARKER**					
GPT2	2.49E-03	1.871	FTH1	2.65E-03	1.272
SLC7A5	2.20E-02	1.473	VLDLR	2.96E-03	1.189
HERPUD1	9.48E-03	1.256	FTL	8.90E-03	1.004
GDF15	2.74E-02	2.787			
ARRDC3	1.35E-03	1.388			
SLC2A1	1.62E-02	1.104			
**SUPPRESSOR**					
LINC00336	1.39E-03	1.084	FTH1	2.65E-03	1.272
PLIN2	2.68E-03	2.864	ENPP2	5.07E-03	1.333
			ACSL3	2.38E-02	0.372
			HIF1A	2.95E-02	1.088

### Functional enrichment analysis of DEGs

**Fig. 3:**
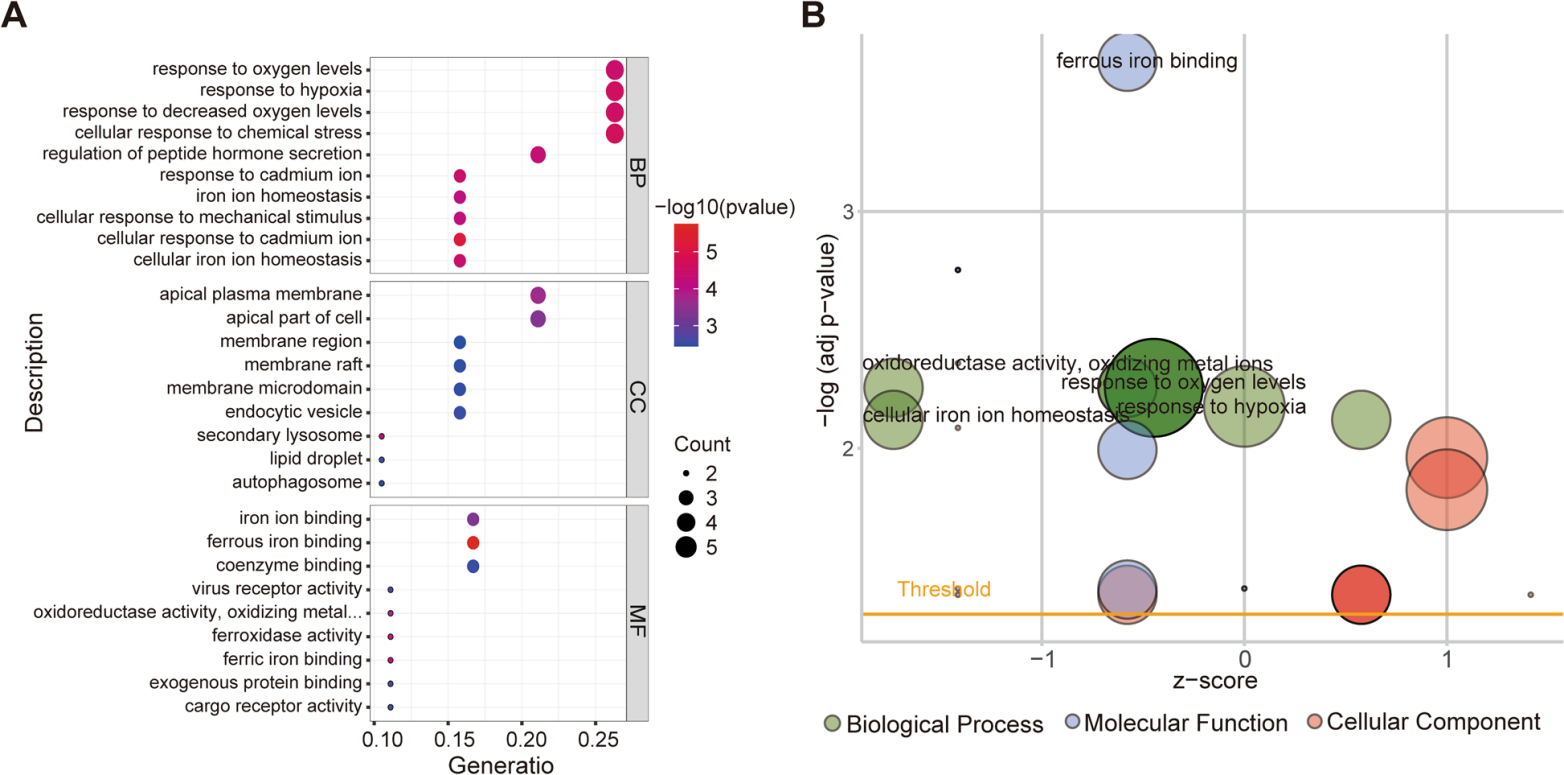
Representative results of GO analyses. **A** Bubble plots of the GO analyses. **B** Results of the GO analyses

To investigate the biological functions and pathways of FRGs in EOPE patients, GO and KEGG enrichment analyses were performed on the 20 genes. The GO analysis showed that differentially expressed FRGs were mainly enriched in hypoxia- and iron-related pathways, such as the response to hypoxia, iron homeostasis and iron ion binding process (Fig. 3A, B and Table S3). The KEGG results showed that the differentially expressed FRGs were closely enriched in central carbon metabolism in cancer, the HIF-1 signaling pathway, necroptosis and ferroptosis (Supplementary Fig. 1).

### PPI network analysis of DEGs

**Fig. 4:**
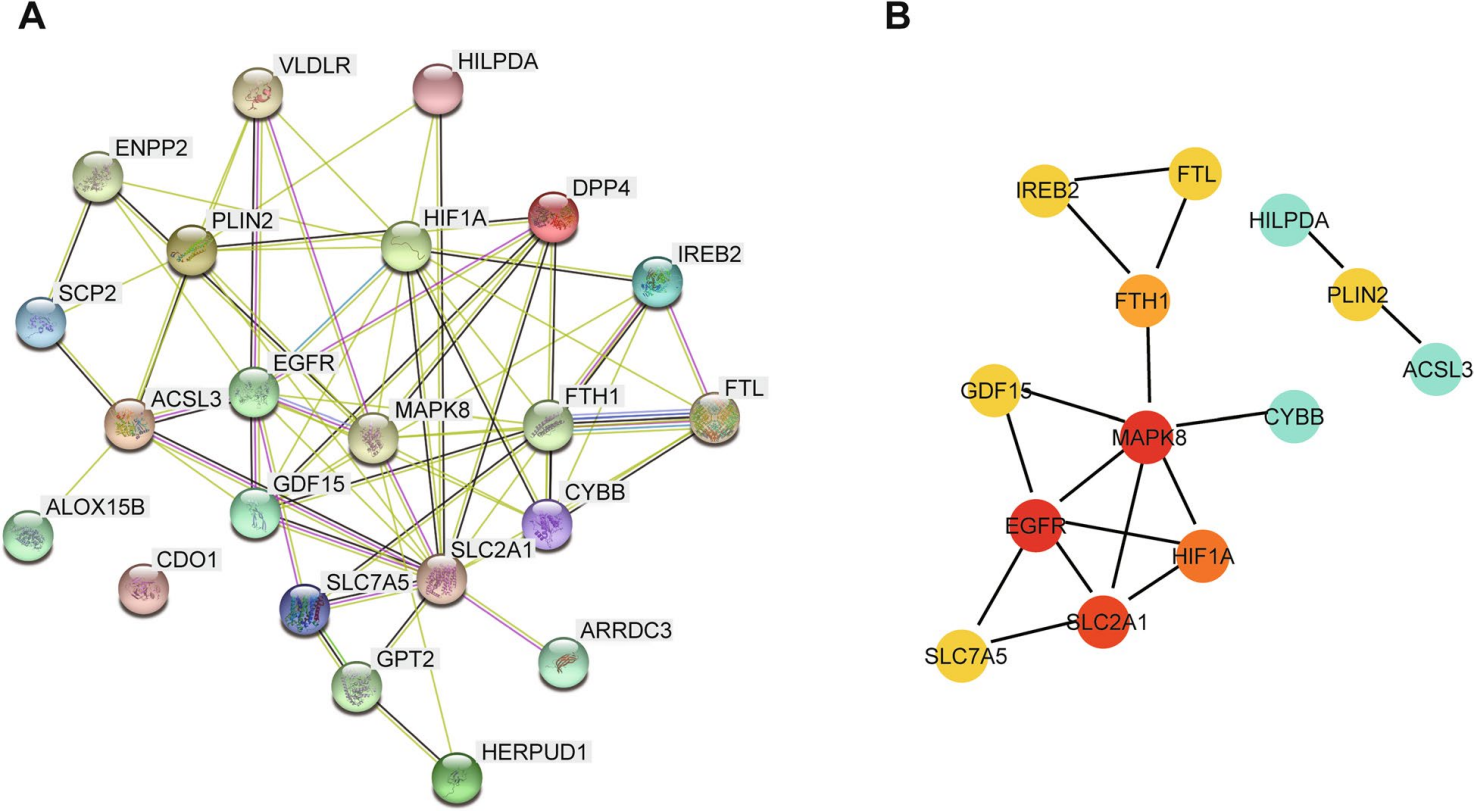
Gene interactions of differentially expressed FRGs in preeclampsia patients. **A** The network of the differentially expressed FRGs downloaded from the STRING database. **B** The top 10 hub FRGs distinguished using color shading from yellow to red, according to the score

The differentially expressed FRGs were analyzed using the STRING online database, and a PPI network with 22 nodes and 66 edges was obtained (Fig. 4A). We used the cytoHubba plugin in Cytoscape to identify the hub FRGs involved in EOPE. As shown in Fig. 4B and Table 2, the top 10 hub FRGs, including mitogen-activated protein kinases 8 (MAPK8), epidermal growth factor receptor (EGFR), solute carrier family 2 member 1 (SLC2A1), hypoxia-inducible factor 1A (HIF1A), ferritin heavy chain 1 (FTH1), growth differentiation factor 15(GDF15), solute carrier family 7 member 5 (SLC7A5), iron responsive element binding protein 2 (IREB2), ferritin light chain (FTL), Perilipin 2 (PLIN2), were identified (Fig. 4B and Table 2).

**Table 2:** Top 10 genes in the network ranked by the degree method

Rank	Name	Score
1	EGFR	10
1	MAPK8	10
3	SLC2A1	8
4	HIF1A	6
5	FTH1	3
6	FTL	2
6	IREB2	2
6	SLC7A5	2
6	GDF15	2
6	PLIN2	2

### Validation of DEGs in PE

**Fig. 5:**
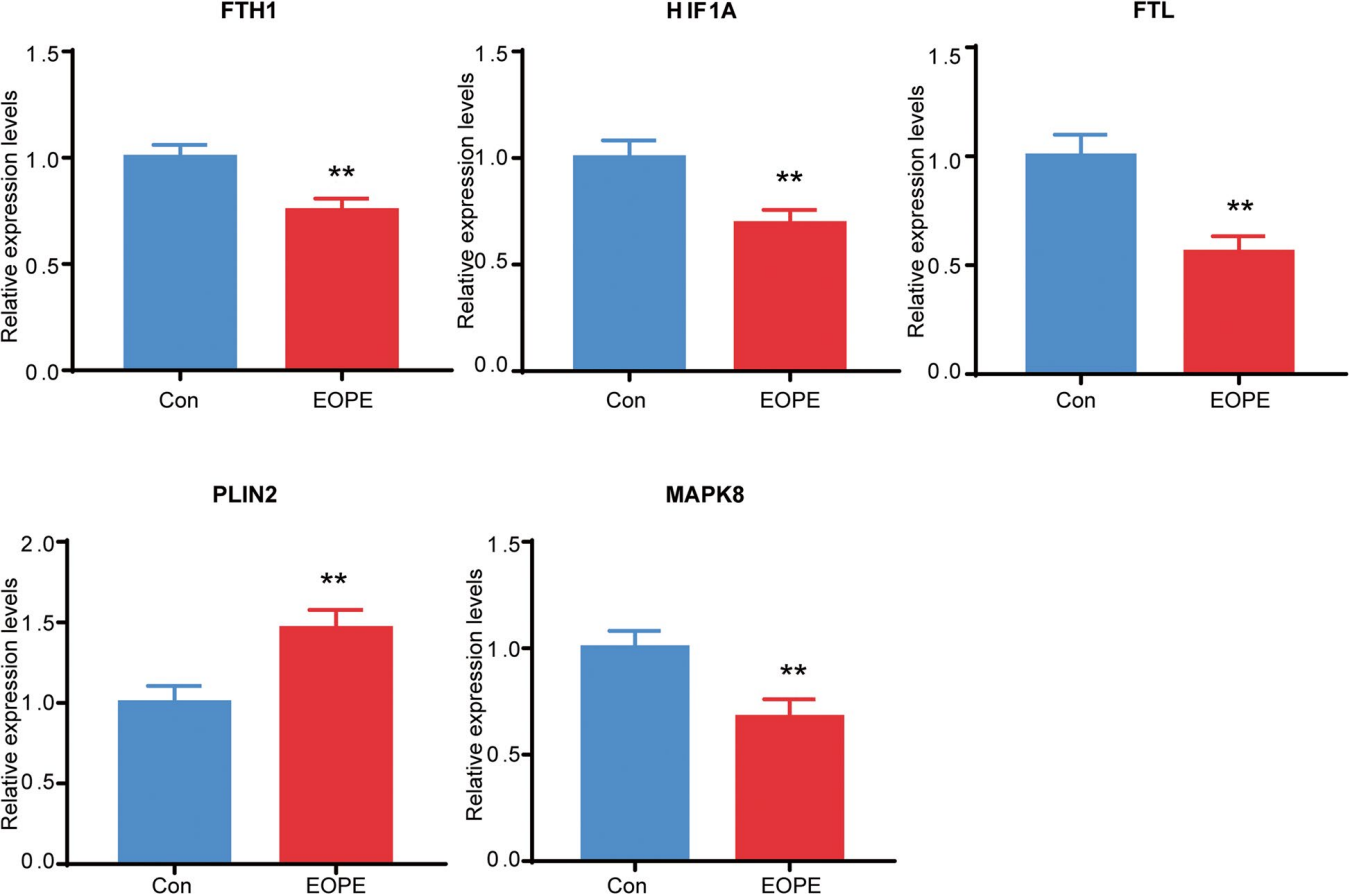
The relative expression of differentially expressed FRGs in the placental samples. The control (Con) group included normal placental samples, and the EOPE group included placental samples from patients with preeclampsia. The sample sizes (Con: *n* = 26, EOPE: *n* = 22) achieve 81–99% power to reject the null hypothesis of equal means using a 2-sided, 2-sample equal-variance t test. **P < 0.01

Placental samples were collected from EOPE (*n* = 22) patients and healthy volunteers (*n* = 26) to validate the 10 hub FRGs using RT-qPCR analysis. The clinical features of the patients are shown in Table 3. As expected, the results showed that the mRNA expression of FTH1, HIF1A, FTL and MAPK8 in the placental samples of EOPE patients was significantly decreased compared with that of healthy controls, while PLIN2 was significantly increased in EOPE patients’ placental samples (Fig. 5).

**Table 3:** Clinical information of the patients

Category	EOPE(*n* = 22)	Normal(*n* = 26)	*P*-value
Age (years)	30.32 ± 1.152	31.96 ± 0.8218	0.2244
Gestational age at delivery (weeks)	33.88 ± 0.5745	38.88 ± 0.2505	< 0.0001
Systolic blood pressure (mmHg)	165.5 ± 3.526	113.1 ± 2.185	< 0.0001
Diastolic blood pressure (mmHg)	108.1 ± 3.097	73.85 ± 1.316	< 0.0001
Proteinuria (%)	100	0	< 0.0001
Neonatal birth weight (g)	2043 ± 181.1	3494 ± 128.9	< 0.0001
1 min Apgar(score)	8.318 ± 0.3443	9.885 ± 0.0639	< 0.0001

## Discussion

Ferroptosis, which is distinct from apoptosis and autophagy, is an iron-dependent programmed cell death initiated by iron-dependent hydroxyperoxidized phospholipids [11]. Oxidative stress and cell damage and death resulting from hypoxia and mitochondrial dysfunction are the major causes of placental pathogenesis in PE [23]. Although ferroptosis has been well characterized in various cancers [24, 25], its role in PE is much less clear. In the present study, we systematically analyzed the expression of ferroptosis genes in the placental samples of patients with EOPE and LOPE. Our results showed that 1) the gene expression profiles in EOPE patients was very different from those in LOPE patients; 2) the significantly different FRGs were mainly involved in EOPE; and 3) the differentially expressed FRGs in EOPE were mainly enriched in hypoxia- and iron-related pathways, such as the response to hypoxia, iron homeostasis and iron ion binding process.

EOPE is often associated with impaired placentation as early as the first trimester, while abnormalities in the maternal vasculature are associated with LOPE. Previous studies have shown that EOPE and LOPE have different gene expression profiles underlying the differential pathogenesis of the two PE subtypes [26, 27]. In this study, we observed similar results. Principal component analysis (PCA) showed that the EOPE subtypes was clustered together and separated from the LOPE subtypes and nonPE samples. The number of DEGs in comparisons of EOPE patients and preterm controls (4215 DEGs) was much greater than that of LOPE patients and term controls (556 DEGs). In addition, only 7 DEGs were found between preterm and term controls, which suggest that gestational age may exert little influence on gene expression. All these results strongly implied the different molecular mechanisms involved in the two clinical subtypes.

There are circumstances that may induce ferroptosis during the development of the placenta, including free iron [28, 29], hypoxia-reoxygenation [30, 31], trophoblastic lipid peroxidation [6, 32] and a failure of the ferroptosis-mitigating guards [33]. Indeed, the potential role of ferroptosis in placental dysfunction and trophoblast injury has been established in recent studies [15–17]. In this study, we systematically analyzed the expression profiles of FRGs in EOPE and LOPE. Interestingly, we found that the differentially expressed FRGs were mainly enriched in EOPE but not in LOPE. Thirty percent of FRGs (6/20), as markers indicating the occurrence of ferroptosis, was upregulated in the placental samples of EOPE patients, while only 10% (2/20) were downregulated. These results implied the great potential roles of ferroptosis in early-onset PE.

The essence of ferroptosis is metabolic cell death that is instigated by the excessive peroxidation of polyunsaturated fatty acids catalyzed by iron [11]. Nonenzymatic lipid peroxidation is essential to initiate the oxidation of polyunsaturated fatty acids [34]. In addition, enzymatic lipid peroxidation, mediated by the lipoxygenase (LOX) family, is another catalyzed chain reaction of polyunsaturated fatty acids [35]. The consequence induced by serial oxidation is the destruction of the membrane, which ultimately results in the occurrence of ferroptosis. Hypoxia-reoxygenation and the production of reactive oxygen species (ROS) commonly occur during implantation and placentation [36, 37]. The accumulation of ROS and lipid peroxidation that results from upregulated levels of oxidative stress is commonly involved in impaired placental function [6]. In addition, iron is rich in placental trophoblasts, even in the case of iron deficiency, because it is actively transferred to the fetus through the placenta [28, 29]. Previous studies have shown that iron imbalance is related to the impaired placental function that characterizes PE [28, 38, 39]. Consistent with this evidence, functional enrichment analysis in the present study revealed that the differentially expressed FRGs in EOPE were mainly enriched in hypoxia- and iron-related reactions. These data support the link between ferroptosis and EOPE that emanates from abnormal implantation and placentation, which highlights the need for further study of the role of ferroptosis in PE and other obstetrical diseases.

In the present study, 10 differentially expressed FRGs were identified as the most significant hub genes. Consistent with the prediction, downregulated genes, including FTH1, HIF1A, FTL and MAPK8, and upregulated PLIN2 were validated by RT-qPCR in EOPE. FTL and FTH1, mainly responsible for iron metabolism, are light and heavy chains of ferritin, respectively. Aberrant expression of the two iron-related genes induces disorder of iron uptake and intracellular storage, which facilitates cell ferroptosis [40]. In particular, FTH1, as a key subunit of ferritin, was reported to be impacted in a variety of biological processes, including regulating immunity [41] and inhibiting apoptosis [42]. HIF1A, as the main transcriptional regulator of the hypoxia response, regulates cell survival in response to stressors. In addition, studies have shown that HIF1A plays an important role in reducing fatty acid β-oxidation and promoting lipid storage [43, 44], which may induce peroxidation-mediated endometrial damage and inhibit ferroptosis [45]. MAPK8 belongs to the family of mitogen-activated protein kinases (MAPKs), which can be activated by environmental stressors to regulate a variety of signaling pathways and play an important role in cell function, from cell survival to cell death [46, 47]. Perilipin 2 (PLIN2), also known as adipogenic differentiation-related protein (ADRP), is wrapped in lipid droplets together with phospholipids and participates in neutral lipid storage in lipid droplets [48]. Recent studies have shown that PLIN2 plays pivotal roles in the regulation of ferroptosis induced by abnormal lipid metabolism in gastric cancer [49].

Taken together, this study provided molecular-level evidence that the two clinical subtypes, EOPE and LOPE, have distinct underlying molecular mechanisms. Importantly, differentially expressed ferroptosis-related genes in EOPE were identified, which provides a link between placental ferroptosis and PE. However, further studies are necessary for deeper inquiry into placental ferroptosis and its role in the EOPE pathogenesis.

## Supplementary Information

**Additional file 1.**

## Data Availability

The datasets generated and/or analysed during the current study are available in the Gene Expression Omnibus (GEO) database (https://www.ncbi.nlm.nih.gov/geo/query/acc.cgi?acc=GSE74341).

## Abbreviations

EOPE: Early-onset preeclampsia
LOPE: Late-onset preeclampsia
PE: Preeclampsia
GO: Gene Ontology
KEGG: Kyoto Encyclopedia of Genes and Genomes
DEGs: Differentially expressed genes
FRGs: Ferroptosis-related genes
PPI: Protein protein interaction
RT–qPCR: Quantitative reverse transcription polymerase chain reaction
PCA: Principal component analysis
