# Correction to: Expression profiles and functions of ferroptosis-related genes in the placental tissue samples of early- and late-onset preeclampsia patients

**DOI:** 10.1186/s12884-022-04512-6

**Published:** 2022-03-23

**Authors:** Nana Yang, Qianghua Wang, Biao Ding, Yingying Gong, Yue Wu, Junpei Sun, Xuegu Wang, Lei Liu, Feng Zhang, Danli Du, Xiang Li

**Affiliations:** 1 Reproductive Medicine Center, Department of Obstetrics and Gynecology, The First Affiliated Hospital of Bengbu Medical College, Bengbu, 233004 Anhui China; 2 Anhui Province Key Laboratory of Immunology in Chronic Diseases, The First Affiliated Hospital of Bengbu Medical College, Bengbu, 233004 Anhui China

**Correction to: BMC Pregnancy Childbirth 22, 87 (2022)**

**https://doi.org/10.1186/s12884-022-04423-6**

In the original publication of this article [1], the authors identified an error in Fig. 3. Supplementary Fig. 1 was uploaded as Fig. 3 by mistake. Correct Fig. 3 is shown below:

**Fig. 3:**
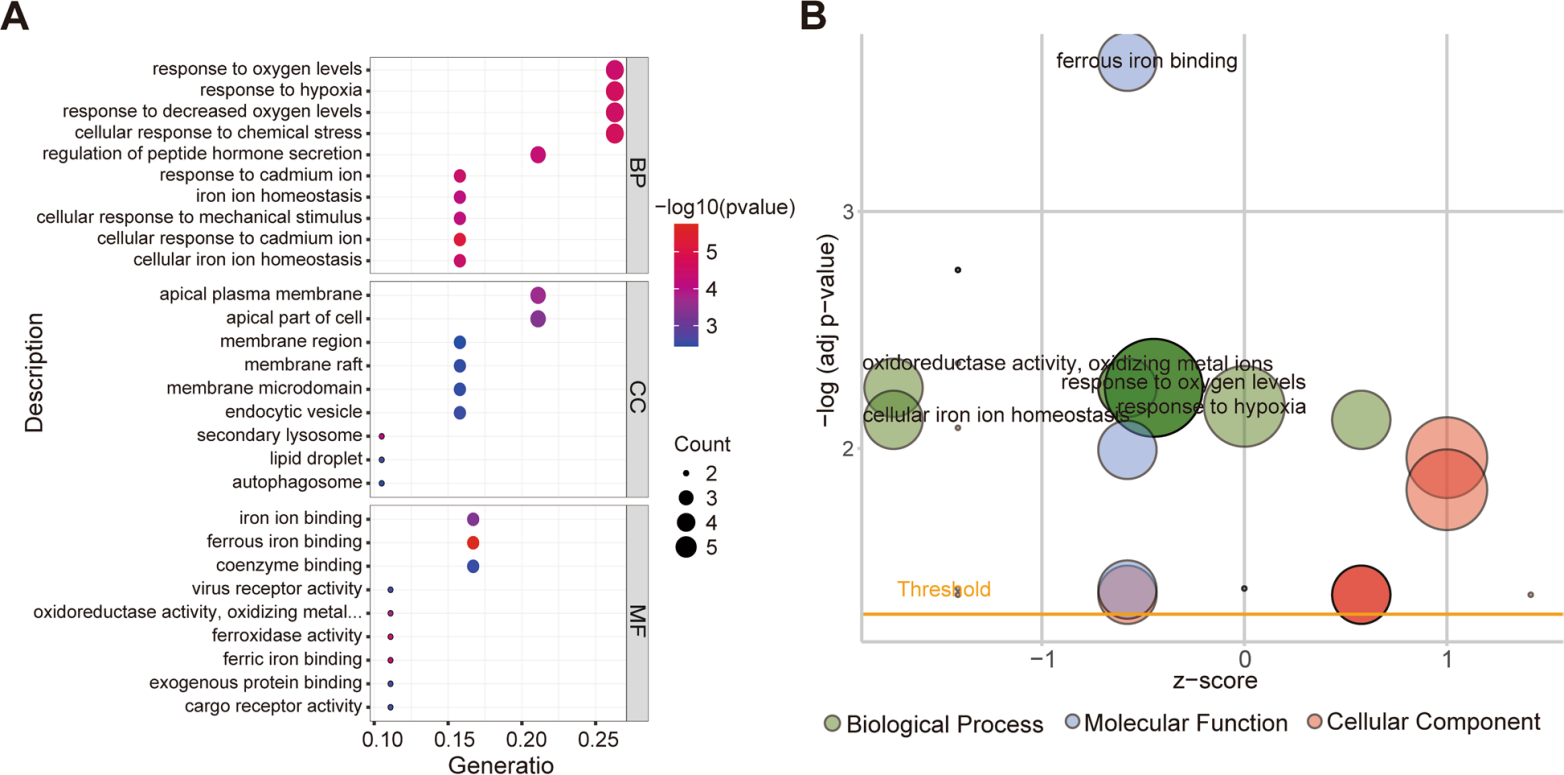
Representative results of GO analyses. **A** Bubble plots of the GO analyses. **B** Results of the GO analyses

The original article has been corrected.
