# Supplementary material for: Expression profiles and functions of ferroptosis-related genes in the placental tissue samples of early- and late-onset preeclampsia patients
Source: BMC Pregnancy Childbirth. 2022 Jan 31;22:87. doi: 10.1186/s12884-022-04423-6 (PMC8805258; doi:10.1186/s12884-022-04423-6)
Supplement: Supplementary file 1 — Additional file 1. [file 12884_2022_4423_MOESM1_ESM.doc]

Supplementary Fig. 1


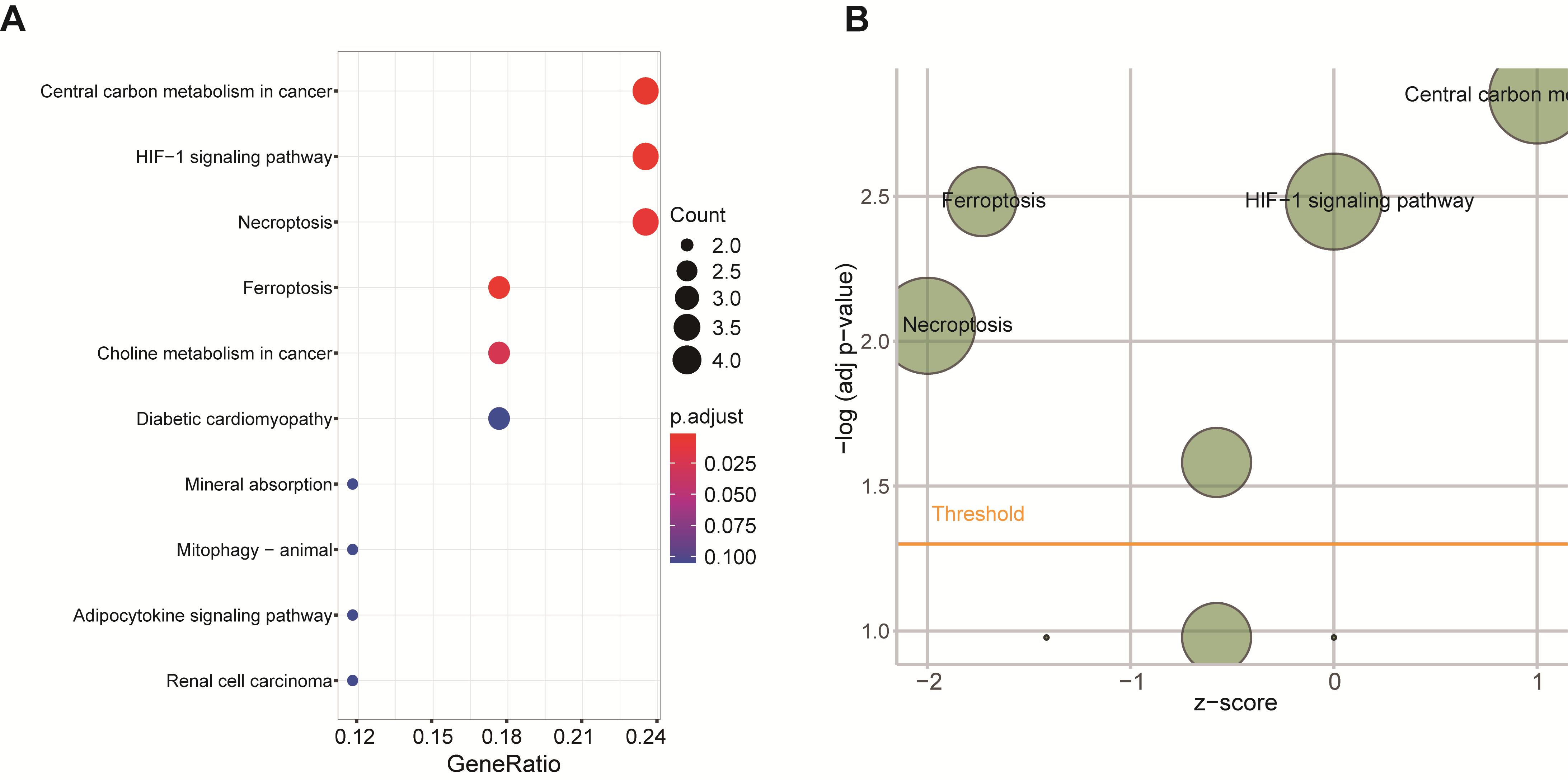


**Supplementary Fig. 1 Representative results of KEGG analyses. (A)** Bubble plots of the KEGG analyses. **(B)** Results of the KEGG analyses.

Table S1. The primer sequences used in qPCR

| **Gene name** | **Primer orientation** | **Sequence** |
| --- | --- | --- |
| EGFR | Forward | 5’-TTGCCGCAAAGTGTGTAACG-3’ |
|  | Reverse | 5’-GAGATCGCCACTGATGGAGG-3’ |
| MAPK8 | Forward | 5’-AGGACTGCAGGAACGAGTTTT-3’ |
|  | Reverse | 5’-TAGCCCATGCCAAGGATGAC-3’ |
| SLC2A1 | Forward | 5’-TGGCATCAACGCTGTCTTCT-3’ |
|  | Reverse | 5’-AGCCAATGGTGGCATACACA-3’ |
| HIF1A | Forward | 5’-ACCTATGACCTGCTTGGTGC-3’ |
|  | Reverse | 5’-GGCTGTGTCGACTGAGGAAA-3’ |
| FTH1 | Forward | 5’-CAGAACTACCACCAGGACTCAG-3’ |
|  | Reverse | 5’-AAGCCACATCATCGCGGTC-3’ |
| FTL | Forward | 5’-AAGGCTCACTCTCAAGCACG-3’ |
|  | Reverse | 5’-AAAGCTGCCTATTGGCTGGA-3’ |
| IREB2 | Forward | 5’-TGGAGAACTAGGCCGAAACTC-3’ |
|  | Reverse | 5’-ACACTGTTTCAGGTTCAGGCA-3’ |
| SLC7A5 | Forward | 5’-GTCAATGGGTCCCTGTTCACATCC-3’ |
|  | Reverse | 5’-GGCGTAGAGCAGCGTCATCAC-3’ |
| GDF15 | Forward | 5’-ATTCGAACACCGACCTCGTC-3’ |
|  | Reverse | 5’-GAGAGATACGCAGGTGCAGG-3’ |
| PLIN2 | Forward | 5’-TGAGATGGCAGAGAACGGTG-3’ |
|  | Reverse | 5’-GCAATTTGCGGCTCTAGCTT-3’ |

Table S2. The ferroptosis related genes

| **Category** | **Ferroptosis-related genes** | |  |
| --- | --- | --- | --- |
| **DRIVER** | | RPL8, IREB2, ATP5MC3, CS, EMC2, ACSF2, NOX1, CYBB, NOX3, NOX4, NOX5, DUOX1, DUOX2, G6PD, PGD, VDAC2, PIK3CA, FLT3, SCP2, TP53, ACSL4, LPCAT3, NRAS, KRAS, HRAS, TF, TFRC, TFR2, SLC38A1, SLC1A5, GLS2, GOT1, CARS1, TP53, ALOX5, KEAP1, HMOX1, TP53, TP53, GLS2, ATG5, ATG7, NCOA4, TF, ALOX5, ALOX12, ALOX12B, ALOX15, ALOX15B, ALOXE3, PHKG2, TFRC, ACO1, IREB2, SLC38A1, GLS2, G6PDX, ULK1, ATG3, ATG4D, ATG5, BECN1, MAP1LC3A, GABARAPL2, GABARAPL1, ATG16L1, WIPI1, WIPI2, SNX4, ATG13, ULK2, NCOA4, ACSL4, TP53, SAT1, ALOX15, ACSL4, LPCAT3, ALOX15, ACSL4, KEAP1, EGFR, NOX4, MAPK3, MAPK1, BID, ACSL4, ZEB1, KEAP1, DPP4, ALOX15, ALOX12, CDKN2A, PEBP1, SOCS1, CDO1, MYB, HMOX1, MAPK8, MAPK9, MAPK1, MAPK3, SLC1A5, CHAC1, MAPK14, LINC00472, NOX4, GOT1, BECN1, PRKAA2, PRKAA1, ELAVL1, BAP1, TP53, ABCC1, ACSL4, MIR6852, ACVR1B, TGFBR1, BAP1, EPAS1, HILPDA, HIF1A, ALOX12, ACSL4, HMOX1, IFNG, ANO6, LPIN1, HMGB1, TNFAIP3, TLR4, NOX4, ATF3, ATM, YY1AP1, EGLN2, MIOX, TAZ, MTDH, IDH1, SIRT1, TAZ, BECN1, FBXW7, PANX1, DNAJB6, BACH1, ACSL4, LONP1 | |
| **MARKER** | | PTGS2, DUSP1, NOS2, NCF2, MT3, UBC, ALB, TXNRD1, SRXN1, GPX2, BNIP3, OXSR1, SELENOS, ANGPTL7, CHAC1, SLC7A11, DDIT4, LOC284561, ASNS, TSC22D3, DDIT3, JDP2, SESN2, SLC1A4, PCK2, TXNIP, VLDLR, GPT2, PSAT1, LURAP1L, SLC7A5, HERPUD1, XBP1, ATF3, SLC3A2, CBS, ATF4, ZNF419, KLHL24, TRIB3, ZFP69B, ATP6V1G2, VEGFA, GDF15, TUBE1, ARRDC3, CEBPG, SNORA16A, RGS4, BLOC1S5-TXNDC5, LOC390705, EIF2S1, KIM-1, IL6, CXCL2, RELA, HSD17B11, AGPAT3, SETD1B, HMOX1, TF, FTL, RPL8, ATP5MC3, TFRC, MAFG, IL33, FTH1, SLC40A1, TF, TFRC, FTH1, GPX4, HAMP, HSPB1, NFE2L2, STEAP3, DRD5, GPX4, DRD4, MAP3K5, MAPK14, SLC2A1, SLC2A3, SLC2A6, SLC2A8, SLC2A12, GLUT13, SLC2A14, EIF2AK4, EIF2S1, ATF4, ALOX5, ALOX12, ALOX15, ALOX5, ACSF2, IREB2, GPX4, HMGB1, HMOX1, NFE2L2, ELAVL1, SLC3A2, SLC7A11, TFAP2C, SP1, HBA1, NNMT, PLIN4, HIC1, STMN1, RRM2, CAPG, HNF4A, NGB, YWHAE, GABPB1, AURKA, MIR4715, RIPK1, PRDX1, MIR30B | |

(continued on next page)

S2 Table. (continued)

| **Category** | **Ferroptosis-related genes** |
| --- | --- |
| **SUPPRESSOR** | SLC7A11, GPX4, AKR1C1, AKR1C2, AKR1C3, GPX4, RB1, HSPB1, HSF1, SLC7A11, GPX4, GCLC, SLC7A11, NFE2L2, SQSTM1, NQO1, HMOX1, FTH1, MUC1, SLC3A2, MT1G, NFE2L2, SLC40A1, SLC7A11, GPX4, SLC7A11, CISD1, SLC7A11, FANCD2, GPX4, NFE2L2, FTMT, HSPA5, ATF4, SLC7A11, GPX4, GPX4, HMOX1, ATF4, NFE2L2, TP53, SLC7A11, HELLS, SCD, FADS2, SRC, STAT3, NFE2L2, PML, MTOR, NFS1, TP63, SLC7A11, TP53, CDKN1A, MIR137, SLC40A1, GPX4, GPX4, ENPP2, VDAC2, FH, CISD2, SLC40A1, MIR9-1, MIR9-2, MIR9-3, CBS, NFE2L2, SQSTM1, GPX4, ISCU, FTH1, ACSL3, OTUB1, CD44, LINC00336, STAT3, BRD4, PRDX6, MIR17, SCD, SESN2, NF2, ARNTL, HIF1A, JUN, CA9, HSPA5, TMBIM4, HSPA5, PLIN2, MIR212, Fer1HCH, AIFM2, AIFM2, LAMP2, ZFP36, GPX4, PROM2, CHMP5, CHMP6, AKR1C1, AKR1C2, AKR1C3, CBS, NFE2L2, CAV1, GCH1 |

Table S3. The enriched terms for different expressed FRGs.

| **Category** | **Term** | **Description** | **Genes** | ***P*-value** |
| --- | --- | --- | --- | --- |
| **BP** | GO:0062197 | cellular response to chemical stress | HIF1A, EGFR, CYBB, MAPK8, SLC2A1 | 5.55E-03 |
|  | GO:0001666 | response to hypoxia | HIF1A, HILPDA, CYBB, DPP4, SLC2A1 | 5.55E-03 |
|  | GO:0036293 | response to decreased oxygen levels | HIF1A, HILPDA, CYBB, DPP4, SLC2A1 | 5.55E-03 |
|  | GO:0006879 | cellular iron ion homeostasis | FTH1, HIF1A, FTL | 5.55E-03 |
|  | GO:0046686 | response to cadmium ion | EGFR, CYBB, MAPK8 | 5.55E-03 |
| **CC** | GO:0005767 | secondary lysosome | FTH1,FTL | 8.19E-03 |
|  | GO:0016324 | apical plasma membrane | EGFR,DPP4,SLC7A5,SLC2A1 | 1.09E-02 |
|  | GO:0045177 | apical part of cell | EGFR,DPP4,SLC7A5,SLC2A1 | 1.49E-02 |
|  | GO:0005811 | lipid droplet | PLIN2,HILPDA | 4.15E-02 |
|  | GO:0030139 | endocytic vesicle | EGFR,CYBB,DPP4 | 4.15E-02 |
| **MF** | GO:0008198 | ferrous iron binding | FTH1,CDO1,FTL | 2.33E-04 |
|  | GO:0004322 | ferroxidase activity | FTH1,FTL | 1.76E-03 |
|  | GO:0016724 | oxidoreductase activity, oxidizing metal ions, oxygen as acceptor | FTH1,FTL | 1.76E-03 |
|  | GO:0008199 | ferric iron binding | FTH1,FTL | 1.76E-03 |
|  | GO:0016722 | oxidoreductase activity, oxidizing metal ions | FTH1,FTL | 4.37E-03 |
